# Compressed Sensing of Extracellular Neurophysiology Signals: A Review

**DOI:** 10.3389/fnins.2021.682063

**Published:** 2021-08-26

**Authors:** Biao Sun, Wenfeng Zhao

**Affiliations:** ^1^School of Electrical and Information Engineering, Tianjin University, Tianjin, China; ^2^Department of Electrical and Computer Engineering, Binghamton University, State University of New York, Binghamton, NY, United States

**Keywords:** compressed sensing, electrophysiology, wireless neural recording, sparse representation (coding), sparse recovery

## Abstract

This article presents a comprehensive survey of literature on the compressed sensing (CS) of neurophysiology signals. CS is a promising technique to achieve high-fidelity, low-rate, and hardware-efficient neural signal compression tasks for wireless streaming of massively parallel neural recording channels in next-generation neural interface technologies. The main objective is to provide a timely retrospective on applying the CS theory to the extracellular brain signals in the past decade. We will present a comprehensive review on the CS-based neural recording system architecture, the CS encoder hardware exploration and implementation, the sparse representation of neural signals, and the signal reconstruction algorithms. Deep learning-based CS methods are also discussed and compared with the traditional CS-based approaches. We will also extend our discussion to cover the technical challenges and prospects in this emerging field.

## 1. Introduction

Extracellular neural recording has been established for decades for monitoring the neuronal ensemble activities with better temporal resolution (Hubel, 1957; Buzsáki, 2004; Stevenson and Kording, 2011). Compared with other neural activity monitoring approaches, extracellular neural recording offers a broad recording spectrum of electrophysiology signals generated by the living brains, which spans from the slow-varying local field potentials (LFPs) to the transient spiking activities (action potentials [APs]) (Buzsáki et al., 2012). Both neural signal modalities carry essential brain processing information and are crucial to the understanding of brain functions. APs play a key role in neuron-to-neuron communication across the entire nervous system and have been widely studied for their functional representation in neural coding, while LFPs reflect the highly dynamic information flows beyond the reach of observing spiking activities from a few neurons, and have been studied for motor decoding in brain–machine interfaces (Andersen et al., 2004), sleep states (Vyazovskiy et al., 2011), sensory processing (Haslinger et al., 2006) as well as higher cognitive processes such as attention, memory, and perception.

To improve the yield of isolated neurons and the spatial coverage of field potentials from extracellular recordings, novel large-scale neural recording technologies with flexible, densely packed microelectrode tips (a few μm in diameter) are highly desired (Berényi et al., 2014; Hong and Lieber, 2019). Such needs motivate the engineering endeavors to create novel large-scale neurotechnologies to meet the scientific and clinical queries for investigating the brain-wide cortical dynamics. Recently, neurotechnologies have emerged with unprecedented recording densities toward cellular resolution, distributed brain regions, and depth structures (Jun et al., 2017; Allen et al., 2019; Musk and Neuralink, 2019; Stringer et al., 2019), which hold tremendous potentials for neuroscientific discoveries. Neuronal population activities can therefore be recorded from diverse brain regions with a high temporal resolution, leading to a better understanding of the functionality of the neural circuits, the information flow, the network connections across different brain regions, and the relationship to the behaviors.

The steep recording density increase leads to the “*Big Neural Data Challenge*.” With tens of kilohertz sampling frequency and more than 10-bit analog-to-digital conversion resolution, several hundreds of megabits per second digitized neural data are generated in real time when incorporating the emerging high-density neural recording probes. The drastically increased neural data pose serious challenges for data processing, storage, and transmission tasks, all of which are of particular relevance to bandwidth-scarce and resource-constrained headstage designs. In general, cable electronics are used to stream an overwhelming amount of neural data online. The power consumption of the data link technologies (Lopez et al., 2017; Intan, 2021) can be non-trivial, and the tethered wire configuration could significantly restrain the envisioned frontier neuroscience research paradigms in many realistic and naturalistic settings. A parallel effort in the community is to augment wireless acquisition capabilities into the neurophysiology experiments, which is of great interest to neuroscientists and brain–machine interface technologists, as chronic brain recording of behaving animals could be achieved in an untethered fashion (Schwarz et al., 2014; Yin et al., 2014; Capogrosso et al., 2016; Zhou et al., 2019; Testard et al., 2021). Novel experimental paradigms can be defined to test hypotheses on the brain functions associated with space coding, foraging, social interaction, and cognitive behaviors for both small (e.g., rodents), large (e.g., swine, non-human primates), and flying animal (e.g., bats) subjects. Bi-directional wireless neural interface devices will also be crucial to the rehabilitation and prostheses for human subjects. Nevertheless, the low data bandwidth and high energy consumption make it challenging to employ the wireless communication components during an electrophysiology study (Larson and Nurmikko, 2016; Nurmikko, 2020).

To ease the challenge, high-fidelity on-chip and on-device neural signal compression schemes (Chae et al., 2008; Gagnon-Turcotte et al., 2016; Wu et al., 2017, 2018; Xu et al., 2018) become essential to relax the bandwidth and energy constraints by reducing the amount of data to be wirelessly transmitted at the system level. For the scope of neural signal compression, several promising approaches have been proposed in the past decades, such as on-chip spike detection and sorting (Lewicki, 1998; Gibson et al., 2011), sparse coding (Kamboh et al., 2007; Gagnon-Turcotte et al., 2016), feature extraction (Wu et al., 2017), and adaptive quantization (Martinez et al., 2018). Moreover, the on-chip hardware overhead for data compression and excessive power consumption cannot be neglected.

Compressed sensing (CS, or compressive sensing) is an emerging signal processing technique for sub-Nyquist sampling and the reconstruction of sparse signals (Donoho, 2006; Candes, 2008). The signal acquisition process of CS is achieved through random/incoherent sampling, while the signal recovery can be achieved using algorithms like convex relaxation or greedy algorithms (Tropp and Gilbert, 2007; van den Berg and Friedlander, 2007; Grant et al., 2008; Zhang, 2009). By its notion, CS performs signal acquisition and compression simultaneously. CS aims to break the Nyquist–Shannon sampling limits, stating that the minimal sampling rate should be at least twice of the signal bandwidth (Oppenheim, 1999). Built upon the assumption of signal sparsity, CS can lead to a much-lowered sampling frequency compared to the Nyquist rate. Since its inception, CS has been widely investigated in many application domains that are sampling speed limited, such as high-speed analog-to-digital converter (ADCs), radio-frequency receivers (Chen et al., 2010; Mishali and Eldar, 2011; Yoo et al., 2012), and magnetic-resonance-imaging (MRI) (Lustig et al., 2008). For emerging wearable and implantable biomedical signal processing applications with limited hardware, power, and data bandwidth, CS has also been demonstrated beneficial for data compression tasks (Chen et al., 2012; Zhao et al., 2018), for its efficient, low-complexity encoder design, while the signal reconstruction can be accomplished offline.

In the past decade, CS has also been actively studied for neural signal compression tasks (Charbiwala et al., 2011; Schmale et al., 2013; Zhang et al., 2015; Sun et al., 2017; Zhao et al., 2018, 2019). CS has several encouraging features that suit neural recording applications. Nevertheless, there are many associated challenges as the neural signals have unique signal characteristics compared to other biomedical signal modalities. This paper aims to provide the audiences a comprehensive review of the past and the current status of CS-based neural recording systems. There are several reviews and surveys on the CS theories and their broad application domains (Craven et al., 2014; Jaspan et al., 2015; Gurve et al., 2020). Nevertheless, there is no review dedicated to the application of CS to the extracellular neural recording scenarios, which will be the primary focus of this paper.

The remainder of this paper is organized as follows. Section 2 covers the background of CS and summarizes the performance metrics that are used throughout the paper. Section 3 reviews the literature on the compressed sensing of the extracellular neural signals. Section 4 reviews deep learning (DL) based CS for neural signals. Section 5 discusses the future challenges and section 6 concludes the paper.

## 2. Background

In this section, we first describe the fundamental theories of CS, including the sensing procedure, the sparsity priors, and the reconstruction algorithms. We then show typical CS-based neural recording system architectures. Finally, we present several quality evaluation criteria for neural signals.

### 2.1. Compressed Sensing

Compressed sensing is an emerging low-rate sampling scheme for the signals known to be sparse or compressible on some basis. The basic CS framework (Donoho, 2006), also called the single measurement vector (SMV) model, can be expressed as

(1) $$\begin{array}{r} \text{y}=\Phi \text{x}+\text{e}, \end{array}$$

where **x** ∈ ℝ^*n*^ is a single-channel signal, **Φ** ∈ ℝ^*m*×*n*^ is the sensing matrix, **e** ∈ ℝ^*m*^ is the measurement noise, and **y** ∈ ℝ^*m*^ is the compressed measurement vector. Usually, Equation (1) is underdetermined, i.e., *m* < *n*, and the ratio *n*/*m* is called the compression ratio (CR).

#### 2.1.1. RIP

To ensure accuracy and robustness for signal recovery using the convex ℓ_1_-norm method, the sensing matrix **Φ** should satisfy the restricted isometric property (RIP) (Candes, 2008) defined as follows:

(2) $$\begin{array}{r} \left(1-\delta_{k}\right)||\text{x}|{|}_{2}\leq ||\Phi \text{x}|{|}_{2}\leq \left(1+\delta_{k}\right)||\text{x}|{|}_{2}, \end{array}$$

where δ_*k*_, the isometry constant of **Φ**, must be smaller than 1. The smaller the value of δ_*k*_, the higher the probability of an exact reconstruction.

#### 2.1.2. Incoherent Sampling

In practice, it is difficult to verify the RIP property, while an alternative approach is to quantify the coherence between the sensing matrix and the sparse dictionary (Candes et al., 2011). The coherence μ between the sensing matrix and the dictionary measures the correlation between any two columns of **Φ** and **Ψ**. Ideally, the coherence will be small, i.e., the two matrices are incoherent, as the value for μ is proportional to the number of the required measurements,

(3) $$\begin{array}{r} \mu \left(\Phi ,\Psi \right)=\sqrt{N}\cdot \underset{1\leq k,j\leq N}{\max}|\langle \Phi_{k},\Psi_{j}\rangle |. \end{array}$$

#### 2.1.3. Synthesis Prior

Since the CS system (1) is underdetermined, the signal **x** cannot be uniquely recovered from the sensing matrix **Φ** and measurements **y**. However, if **x** has a *synthesis sparse prior* (SSP) (Bruckstein et al., 2009), i.e.,

(4) $$\begin{array}{r} \text{x}=\Psi \theta , \end{array}$$

where **Ψ** ∈ ℝ^*n*×*n*^ is a pre-defined dictionary, and the signal's representation ***θ*** ∈ ℝ^*n*^ is assumed to be *s*-sparse, i.e.,

(5) $$\begin{array}{r} ||\theta |{|}_{0}≜|\text{supp}\left(\theta \right)|=s\ll n, \end{array}$$

or is well approximated by an *s*-sparse vector, then it is possible to estimate **x** via

(6) $$\begin{array}{r} \hat{x}=\Psi \hat{\theta }\;\text{with }\hat{\theta }=\arg\underset{\theta }{\min}||\text{y}-\Phi \Psi \theta |{|}_{2}^{2}+\lambda f\left(\theta \right), \end{array}$$

where *f*(·) is a regularization function, and λ is a regularization parameter. A widely used penalty is the ℓ_1_-norm penalty, namely *f*(***θ***) = ||***θ***||_1_ (Becker et al., 2011).

Motivated by many applications such as EEG/MEG source localization and DOA (direction of arrival) estimation, where multi-channel signals are measured simultaneously, the SMV model (1) has been extended to the multiple measurement vector (MMV) model in Cotter et al. (2005), given by

(7) $$\begin{array}{r} \text{Y}=\Phi \text{X}+\text{E}, \end{array}$$

where **X** ∈ ℝ^*n*×*l*^ is an *l*-channel signal, **Φ** ∈ ℝ^*m*×*n*^ is the sensing matrix, **E** ∈ ℝ^*m*×*l*^ is the measurement noise, and **Y** ∈ ℝ^*m*×*l*^ is the compressed data. An essential assumption in the MMV model is that the support (i.e., indexes of nonzero entries) of every column in **X** is identical. Therefore, **X** is simultaneous sparse (also referred to as row-sparse), i.e., a few rows of **X** are nonzero rows. Similar to (6), the estimate of **X** is given by

(8) $$\begin{array}{r} \hat{X}=\Psi \hat{\Theta }\;\text{with }\hat{\Theta }=\arg\underset{\Theta }{\min}||\text{Y}-\Phi \Psi \Theta |{|}_{F}^{2}+\lambda g\left(\Theta \right), \end{array}$$

where ||·||_*F*_ denotes the Frobenius norm (ℓ_2_-norm of all the elements) of the matrix and *g*(·) is a regularization function encouraging the simultaneous sparsity. One popular penalty is the ℓ_2,1_-norm penalty (Ding et al., 2006), namely $g\left(\Theta \right)=\overset{n}{\underset{i=1}{\sum }}||\Theta_{i,\cdot }|{|}_{2}$. In (8), **Θ** is assumed to be row-sparse.

#### 2.1.4. Analysis Prior

While the signal reconstruction with SSP has been extensively studied, constructing an appropriate sparse dictionary remains challenging when using CS for neural signal compression. The neural signal segments are non-sparse on widely used dictionaries such as discrete Fourier transform (DFT) basis and discrete cosine transform (DCT) basis. Signal reconstruction using these dictionaries will severely reduce the accuracy. Moreover, the non-stationary behaviors of neural signals pose practical issues over the synthesis model-based methods, and the joint sparsity assumption is valid for only a minor portion of the MMV model. To overcome this limitation, an *analysis sparse prior* (ASP) that takes an analysis point of view has been proposed by Elad et al. (2007). For a signal of interest, ASP assumes that the analysis coefficient vector

(9) $$\begin{array}{r} \text{z}=\Omega \text{x} \end{array}$$

is expected to be sparse, where **Ω** ∈ ℝ^*d*×*n*^ denotes a redundant analysis operator (*d* ≥ *n*), and ρ = *d*/*n* is the redundant ratio. Note that for an invertible square dictionary, SSP and ASP are the same with **Ψ** = **Ω**^−1^ (Elad et al., 2007). While ASP seems similar to the synthesis counterpart, it is very different when dealing with a redundant operator *d* > *n* (Nam et al., 2013). With ASP, the optimization problem for SMV signal recovery can be formulated as

(10) $$\begin{array}{r} \hat{x}=\arg\underset{\text{x}}{\min}||\text{y}-\Phi \text{x}|{|}_{2}^{2}+\lambda f\left(\Omega \text{x}\right). \end{array}$$

Similarly, the optimization problem for MMV signal recovery is given by

(11) $$\begin{array}{r} \hat{X}=\arg\underset{\text{X}}{\min}||\text{Y}-\Phi \text{X}|{|}_{F}^{2}+\lambda g\left(\Omega \text{X}\right). \end{array}$$

The regularization functions *f*(·) and *g*(·) are defined in (6) and (8). The successful recovery of the original signals from the compressed measurements using (10) and (11) has been theoretically guaranteed under the restricted isometry property adapted to the dictionary (D-RIP) and restricted orthogonal projection property (ROPP) (Candes et al., 2011; Nam et al., 2013; Peleg and Elad, 2013).

### 2.2. CS-Based Neural Recording System Architecture

**Figure 1:**
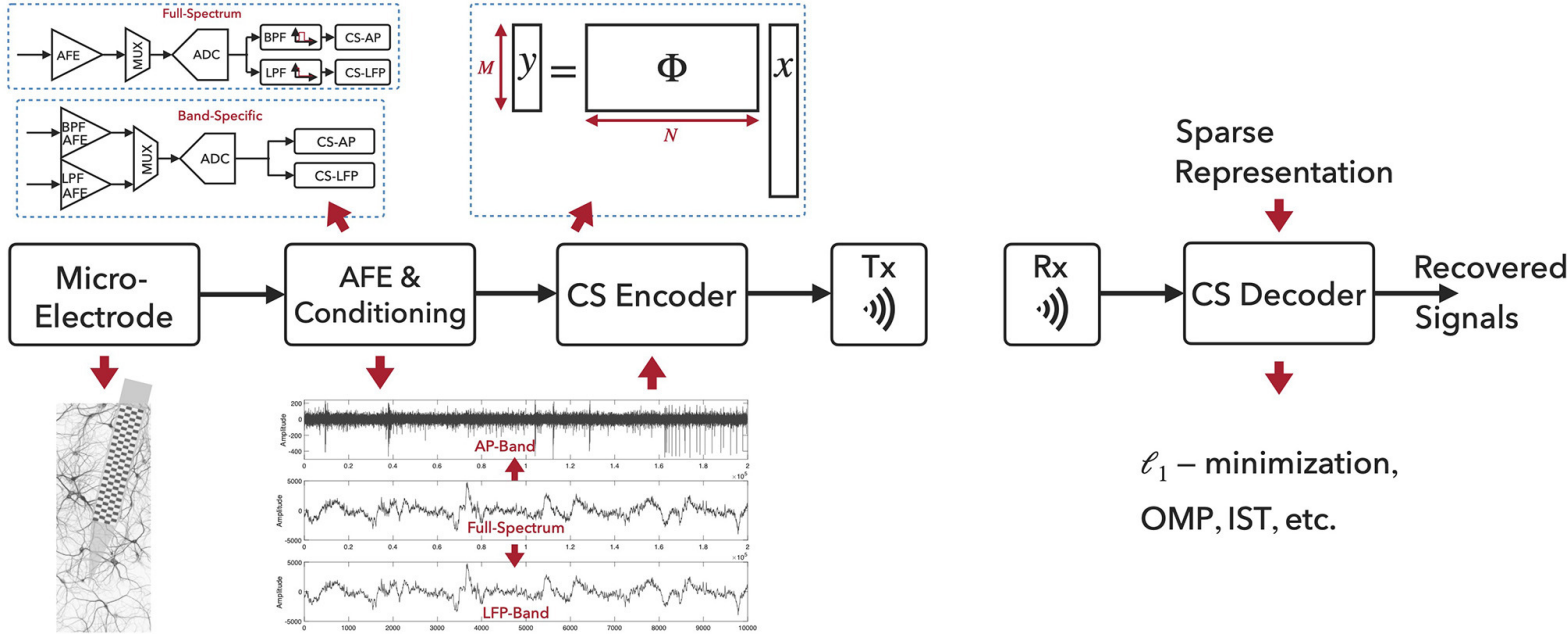
Compressed sensing based neural recording architecture and signal processing flow.

Figure 1 illustrates the CS-based neural recording system architecture and the signal processing flow. The aggregated extracellular neural signals are first picked up by the micro-machined neural probes. The recorded signals from brain structures contain broadband components up to tens of kilohertz, while the signals of general interest lie in two distinct frequency bands, i.e., the local field potentials located in the low-frequency band (below 500 Hz) and the action potentials in the high-frequency band (a few kilohertz). However, full-spectrum neural signals are not sparse on commonly used bases, and the sampling and compression of raw neural signals using CS would severely degrade the reconstruction performance. One widely used method to improve the neural signal reconstruction performance is to filter the full-spectrum signals into APs and LFPs for the separate compression tasks. Two types of neural recording architectures exist, as depicted in Figure 1. In the *Full-Spectrum* type, the broad-band neural signals are acquired and conditioned via analog front-end (AFE; e.g., neural amplifiers) with appropriate signal amplitude and bandwidth, and then digitized via Nyquist-rate ADCs. Digital band-pass filters (BPF) and low-pass filters (LPF) are employed and CS will then be applied on the filtered AP and LFP band signals. In the *Band-Specific* type, the neural signals are first filtered in the analog domain and then digitized. CS will be applied after the digitization. When applying CS to AP signals, CS is often combined with spike detection approach (Zhang et al., 2015; Liu et al., 2016; Sun et al., 2017; Wu et al., 2018; Zhao et al., 2018, 2019) for further compression. For AP signals, the neural spike events are detected and aligned temporally to their absolute peaks. The aligned segments (e.g., a segment of 64 samples) containing the spikes are then compressed via the CS technique. For LFP signals, CS can be directly applied to the time-series LFP data (e.g., with a segment of 256/512 samples).

### 2.3. Quality Evaluation Criterion

To evaluate the performance of CS recovery, the most widely used metric is the signal to noise and distortion ratio (SNDR), which quantifies the error between the original neural signal **x** and the reconstructed signal $\hat{\text{x}}$:

(12) $$\begin{array}{r} \text{SNDR}=20\log\frac{||\text{x}|{|}_{2}}{||\text{x}-\hat{\text{x}}|{|}_{2}}. \end{array}$$

SNDR is often used to evaluate the segment-to-segment error. For LFP reconstruction, SNDR can also be used to evaluate the spectral feature reconstruction capability across widely adopted frequency bands including δ and θ (1–8 Hz), α (8–12 Hz), β (12–30 Hz), γ and high-γ bands (30–70, 70–150 Hz), respectively. The other two widely used metrics are to evaluate the reconstruction accuracy in the frequency domain. The first metric is the average absolute error of spectral power (ϵ_SP_), defined as

(13) $$\begin{array}{r} \epsilon_{\text{SP}}=\frac{1}{\#\text{Seg}}\overset{\#\text{Seg}}{\underset{i=1}{\sum }}|{\text{SP}}_{\text{rec}}\left(i\right)-{\text{SP}}_{\text{ori}}\left(i\right)|, \end{array}$$

where #Seg is the total number of LFP segments, SP_ori_(*i*) is the ground-truth of spectral power in the *i*th segment calculated from the original LFPs, and SP_rec_(*i*) is the spectral power calculated from the recovered LFPs. The second metric is the average absolute error percentage of spectral power (ϵ_PSP_), defined as:

(14) $$\begin{array}{r} \epsilon_{\text{PSP}}=\frac{1}{\#\text{Seg}}\overset{\#\text{Seg}}{\underset{i=1}{\sum }}\frac{\left|{\text{SP}}_{\text{rec}}\left(i\right)-{\text{SP}}_{\text{ori}}\left(i\right)\right|}{{\text{SP}}_{\text{ori}}\left(i\right)}, \end{array}$$

Besides the aforementioned metrics, classification accuracy (CA) is also widely used to evaluate the spike sorting performance of neural action potentials. The CA was defined as:

(15) $$\begin{array}{r} \text{CA}=\frac{\#\text{correctly classified APs}}{\#\text{total APs}}\times 100\%. \end{array}$$

To compute CA for a given method, all APs were compressed and reconstructed, then principal component analysis (PCA) was used to extract features from reconstructed APs. Finally, the first three principal components of each AP were used by clustering algorithms such as superparamagnetic clustering (SPC) (Quiroga et al., 2004), and the classification results were compared with the ground truth labels.

## 3. Compressed Sensing of Extracellular Neural Signals

In this section, we review the application of CS to extracellular neural signal compression. We first introduce the CS encoding part, which includes the sensing matrix design and hardware architecture. We then describe the sparse representations of both APs and LFPs. Table 1 summarizes the reviewed literature on the compressed sensing of neural signals in a chronological order, with implementation details on CS encoding and decoding processes.

**Table 1:** Comparison of compressed sensing (CS) methods for neural signals.

**References**	**CS encoder**	**CS decoder**	**Dataset**	**SNDR/CR**
Gangopadhyay et al. (2011)	Analog	Wavelet	Physiobank	NA
	Bernoulli	ℓ_1_-min	ECoG, EEG	16x
Charbiwala et al. (2011)	NA	Learned supports	Private	≈20 dB
		BPDN	EEG	2x
Chen et al. (2012)	Digital	NA	Private	≈10 dB
	Bernoulli/Binary	ℓ_1_-min	EEG	10x
Schmale et al. (2013)	NA	DWT/DCT/DFT/WHT	MT dataset	≈20 dB
		NA	Spikes, LFPs	2x
Zhou et al. (2013)	NA	K-SVD	Hippocampus	≈13.7 dB
	Circulant and Toepliz	IST	Spikes	60x
Suo et al. (2014)	Digital	Spike data	Leicester	≈20 dB
	Bernoulli	IST	Spikes	3x
Shoaran et al. (2014)	Analog	Gabor	Private	≈14 dB
	Bernoulli	ℓ_1, 2_-min	ECoG	16x
Zhang et al. (2014)	Digital	K-SVD	Leicester	≈13 dB
	Bernoulli	OMP	Spikes	≈5x
Zhang et al. (2015)	Digital	K-SVD	Private	≈13 dB
	Bernoulli	OMP	Spikes	16x
Zamani et al. (2016)	Digital	BK-SVD	Leicester	≈18 dB
	Bernoulli	BSBL	Spikes	9.8x
Sun et al. (2017)	Digital	Analysis prior	Leicester	≈12 dB
	Bernoulli	ℓ_1_-min	Spikes	8x
Zhao et al. (2018)	Digital	K-SVD	HC-1, CHB-MIT	≈12 dB
	Sparse Binary	ℓ_1_-min	Spikes, EEG	4x
Zhao et al. (2019)	Digital	DCT	HC-1	≈28/25 dB
	Binary	Binary weighted ℓ_1_-min	Spikes, LFPs	2x
Sun et al. (2021a)	Digital	Untrained DNN	Leicester, Neuropixels	≈16 dB
	Gaussian		Spikes	5x
Sun et al. (2021b)	Digital	ADMM	PFC-2, Neuropixels	≈18 dB
	Sparse Binary		LFPs	32x

### 3.1. CS Encoding: Sensing Matrices and Hardware Architecture

One unique advantage of CS is that the sensing matrices can be efficiently constructed and implemented in hardware. In practice, sensing matrices with binary entries have been widely investigated with algorithmic performance guarantees and efficient hardware implementation for both analog and digital CS encoders.

Gangopadhyay et al. (2011) presented an analog-domain CS front-end for sub-Nyquist sampling sparse brain signals like electroencephalogram (EEG) and electrocorticogram (ECoG). A one-bit switched-capacitor multiplying digital-to-analog (MDAC)/summation circuit was proposed for CS acquisition, which adopts a Bernoulli random matrix as the sensing matrix. Shoaran et al. (2014) introduced an area- and power-efficient multichannel analog CS encoder architecture for iEEG/ECoG signals for seizure prediction. This work exploits the spatial sparsity of the signals recorded from an ECoG electrode array. The benefits of employing a multichannel CS scheme were validated analytically and experimentally in a 0.18 μm CMOS process. The results of simulations and subsequent reconstructions show the possibility of recovering fourfold compressed intracranial EEG signals with an SNDR up to 21.8 dB while consuming 10.5 μW of power within an effective area of 250 × 250 μm per channel.

Chen et al. (2012) presented a comparative analysis between the analog and digital implementations of CS encoder designs. For biomedical signals that are not sampling frequency limited, digital CS encoders are more advantageous compared to the analog counterpart at the system level even when a Nyquist-rate ADC is used before the CS encoding stage. Binary entry sensing matrices like Bernoulli (±1) or random binary (0/1) matrices are adopted for digital CS encoder implementations, which avoids the hardware-demanding multipliers. As such, for the scope of extracellular neural signals, the majority of CS encoding stages are implemented in digital styles. A parallel CS encoder with Bernoulli random matrix was presented by Zhang et al. (2014), where a 25-channel CS encoder occupies 0.06 mm^2^ silicon area in a 0.18 μm CMOS process and dissipates 270 nW power consumption with a 20 kHz sampling frequency and a supply voltage of 0.6 V. To further reduce the CS encoder hardware toward high-density on-chip neural signal compression tasks, the optimization of sensing matrices has been considered using both deterministic and random sparse sensing matrices (Zhao et al., 2016, 2018). A class of deterministic measurement matrices, namely a Quasi-Cyclic Array Code (QCAC) based matrix and a class of random measurement matrices, termed (1,s)-sparse random binary matrix [(1,s)-SRBM] were constructed. Both types of matrices are highly sparse and constructed with binary entries (0/1), which are exploited to realize both area- and energy-efficient CS encoder VLSI architectures. Note that both types of sensing matrices have sparse column features and can be efficiently generated online, thereby considerably reducing the needed amount of adders in traditional parallel CS encoder designs (Chen et al., 2012).

### 3.2. CS Decoding: Sparse Representation and Recovery for Neural Signals

To achieve successful CS recovery, the signal of interest should satisfy the sparsity assumption. A major task of successful CS recovery is the proper design of sparsifying dictionaries/bases. Nevertheless, it is often challenging to find the proper sparse representations of the real-world neural signals on the decoder side. Moreover, there is no consensus and understanding of which sparsifying dictionary will be suitable for neural signals as they often exhibit non-sparse structures in most known bases like wavelet, Gabor, or Fourier types (Charbiwala et al., 2011; Gangopadhyay et al., 2011; Schmale et al., 2013; Shoaran et al., 2014). Moreover, neural signals are non-stationary and highly dynamic. In a multichannel neural recording setting, spike events are temporally sparse and the firing rates can vary significantly across different recording channels. Additionally, spike shape variation is common during chronic *in vivo* recording experiments, which might be caused by electrode drifts, oxidation from electrode-electrolyte interaction, sparse-firing neurons, or time-varying variations from the same neuron. These facts further complicate the discoveries of sparse representation of neural signals. In this section, we will separately discuss the sparse representation of different types of neural signals.

#### 3.2.1. Sparse Representation and Recovery for APs

A large body of research has focused on the compression of AP signals, which are the most informative biomarkers of interest for distinguishing the firing events from different neurons via spike sorting (Lewicki, 1998; Gibson et al., 2011). Bulach et al. (2012) considered applying CS for the compression of running AP band signals on both synthetic and recorded neural spike signals. Both wavelet (level-6, db-8) and learned dictionaries are used for signal recovery. Nevertheless, the authors argued that CS is generally *not* recommended for compressing AP band neural signals, whereas CS is still applicable to the extracted and aligned spikes for further data rate reduction. Schmale et al. (2013) studied the common sparsity bases (Discrete Fourier/Cosine/Wavelet and Walsh-Hadamard Transforms) for both APs and LFPs. The authors argued that discrete cosine transform (DCT) turns out to be best suited for both neural signal modalities.

Although there is a limited success on the compression rate and the recovery performance using the known sparse basis with standard CS recovery methods, a data-dependent sparse basis is further investigated to improve the signal recovery performance (Charbiwala et al., 2011; Suo et al., 2013, 2014; Zhou et al., 2013; Zhang et al., 2014, 2015). Charbiwala et al. (2011) investigates the compression of detected neural spikes, where the authors assumed empirically that spikes from different neurons are compressible in the wavelet domain (i.e., Daubechies wavelet) and exhibit nearly identical sparsity supports. These identical wavelet supports were learned over time to form a union of supports for spike recovery. The reconstruction algorithm is a combination of both BPDN (Chen and Donoho, 1994) (Basis Pursuit DeNoising) and modified BPDN (Lu and Vaswani, 2010) to leverage the union of supports. A 20-dB spike recovery SNDR and over 90% spike CA can be obtained for a compression ratio of 2. Zhou et al. (2013) demonstrated a sparsifying basis design via dictionary learning approach K-SVD (Aharon et al., 2006) to construct an overcomplete dictionary for the detected and aligned neural action potentials. Iterative shrinkage thresholding (IST) algorithm was adopted for signal reconstruction. The learned sparsifying dictionary outperforms the DWT-based one with a compression ratio of 5 for an SNDR of 13.7 dB. Suo et al. (2014) introduced a multi-mode compressed sensing system for implantable neural recordings. Exploiting the *self expressiveness*, the authors proposed to use the spike data directly as the sparsifying dictionary. This choice of sparsifying dictionary shows comparable performance to the trained signal-dependent dictionary without extra computation for dictionary learning. Zhang et al. (2014) proposed a compact compressed sensing system for implantable neural recording applications, which also exploits K-SVD for sparse dictionary learning. The signal recovery can be achieved with a coarse estimation of spike shapes based on the learned sparse dictionary, and a fine detail estimation using a wavelet dictionary. Band-limited, inter-spike signals can be recovered via a standard CS recovery method using DWT as sparsifying bases. In Zamani et al. (2016), the block K-SVD (BK-SVD) algorithm was employed to train a block-sparsifying dictionary for neural spikes, then the block sparse Bayesian learning (BSBL) algorithm was adopted to reconstruct the original spikes. The proposed method achieved an overall CR of ~11.6 and an SNDR that was up to 8 dB higher than that obtained without spike detection.

Note that signal-dependent sparse representation for neural signal compression cannot adapt to spike shape changes and thereby suffer from reconstruction quality loss when neural signals show varied sparsity supports over time. To accommodate this issue, Zhang et al. (2015) presented a low-power, closed-loop CS neural recording system, where a quality evaluation (QE) block was augmented to provide closed-loop feedback for reconstruction quality estimation and retraining. At the decoder side, the authors argued to adopt measurement SNDR_**y**_ ($20\log_{2}\frac{||\text{y}|{|}_{2}}{||\text{y}-\hat{\text{y}}|{|}_{2}}$) as a quality estimation metric, where **y** is the received measurement, and $\hat{\text{y}}$ is the reconstructed measurement obtained after the signal recovery ($\hat{\text{y}}=\Phi \hat{\text{x}}$). A linear correlation is observed between SNDR_**y**_ and SNDR_**x**_. Therefore, when SNDR_**y**_ is lower than a threshold, a reduced recovery quality is observed and re-training is needed to improve the signal recovery. This quality estimation is performed on the decoder side, thereby posing no extra cost over the on-chip encoder resources.

Recently, a few online methods have been proposed for data-independent CS recovery of neural signals, which can eliminate the training and updating procedures required in the data-dependent approaches. The analysis sparse model has been employed for CS-based neural recording (Sun et al., 2017). The analysis model was adopted to enforce sparsity of the neural signals, overcoming the drawbacks of conventional synthesis models and enhancing the recovery performance. A multi-fractional-order difference matrix was constructed as the analysis operator and a group weighting analysis ℓ_1_-minimization was proposed for signal recovery. Experimental results on both the synthetic and real datasets revealed that the proposed approach outperforms data-dependent CS-based methods in terms of both spike recovery quality and CA. The synthesis sparse model has also been re-visited to enhance the CS reconstruction performance (Zhao et al., 2019) by exploiting additional neural signal structure priors, such as BPF pole and the corner frequency information. A binary-weighted ℓ_1_-minimization algorithm was proposed to enforce the block structure by adding extra penalties to the sparse solvers when projections were made to high-frequency atoms, and improved the recovery performance for both AP and LFP signals, respectively.

#### 3.2.2. Sparse Representation and Recovery for LFPs

Unlike AP signals, the compressed sensing of LFP signals only started to be addressed recently. Schmale et al. (2013) conducted a preliminary sparsity level study for the application of CS to the joint-compression of both LFPs and APs. Four well-known linear transformations (discrete Fourier/cosine/wavelet transform and Walsh–Hadamard transform) have been investigated and compared. Based on the sparsity analysis approach, the authors argued that the discrete consine transform (DCT) turned out to be best suited for both neural signal modalities. Nevertheless, detailed CS performance has not been performed. Zhao et al. (2019) introduced a coarse-grained approach for LFP signals, where a high-γ corner frequency is used as the block boundary for using a binary-weighted ℓ_1_-minimization algorithm. Based on the analysis model, CS reconstruction performance for LFPs was further improved by adopting the simultaneous sparse prior (Sun et al., 2021b) for joint-sparse LFP signals. The proposed method reinforces the sparsity with an optimal continuous order difference matrix as the analysis operator. A non-convex optimizer with an alternating direction method of multipliers (ADMM)-based solver was proposed to recover LFPs accurately and fast, which is promising for large-scale neural recording systems.

### 3.3. Discussion

As illustrated in Table 1, Bernoulli and binary sensing matrices are the dominant hardware implementation choices in most previous reported literature. Another observation is that the analog-domain CS encoder has limited applications to the extracellular neural signals. This is in part caused by the hardware implementation overhead as compared to the digital implementation, and the lack of knowledge on the sparse representation of complex neural signal modalities, including full-spectrum, LFP-, and AP-band signals in the previous literature. Note that the hardware cost of CS encoder is proportional to the segment size *n*, therefore the current CS encoder designs may not scale well toward large-scale neural recording applications. In fact, it is still possible to apply the analog CS encoding scheme after the analog filters, as long as the proper sparse priors are used during the signal reconstruction. Both synthesis (SSP) and analysis (ASP) sparse priors have been studied to improve the neural signal reconstruction quality. Early efforts have primarily focused on SSP and the corresponding recovery algorithms with rigorous theoretical guarantees (e.g., ℓ_1_-minimization and OMP). The common drawback, however, is the poor reconstruction performance caused by non-sparse nature of the neural signals. As a result, learning sparse representations for neural signals has been proposed to improve reconstruction quality. However, this causes new issues regarding the model robustnes s and practical deployment. Recent efforts on the analysis model and the spectral-sparse synthesis model have provided high-quality and training-free CS schemes well suited for both LFPs and APs. Finally, current CS methods for AP compression is still limited. First, overlapping spikes are precluded in the existing literature. It is also worth noting that spike alignment after detection is pre-assumed when applying CS to neural spike signals, which may cause additional hardware overheads to ensure successful CS recovery.

## 4. Deep Learning as a Framework to Solve Inverse Problems in CS

There are a number of efforts employing the DL methods for CS-based neural signal compression tasks. Here, we provide a brief overview on how deep neural networks (DNNs) can be used to estimate $\hat{x}$ for a given measurement *y* by learning a statistical signal transformation. DL can leverage large amounts of neural data to learn statistical transformations in certain high-dimensional spaces to improve the conventional CS algorithms and achieve superior results. The compression and reconstruction of APs (Sun et al., 2016; Sun and Feng, 2017; Wu et al., 2018) have been studied using DL. Nevertheless, there has been little literature on the LFPs compression using DL.

As a regression task, DL learns to solve the inverse problems in CS through supervised learning (Hecht-Nielsen, 1992). After training, no additional hyperparameter tuning is required. This stands in contrast to the traditional reconstruction algorithms in CS, where hyperparameters (e.g., sparsity in OMP and noise level in BPDN) need to be carefully hand-tuned, often through an iterative process to achieve the optimal convergence. By performing the desired transformation in a single feedforward manner, DL outperforms traditional iterative methods in terms of inference speed in general. Different types of DL models have been studied. Considering that AP is a time series signal, early works used fully connected (FC) layers to compress and reconstruct AP. Sun et al. (2016) and Sun and Feng (2017) presented a multilayer perceptron for CS, in which a reconstruction network consisting of multiple FC layers was optimized. Traditional CS used random sensing matrices (whose entries were randomly drawn from Gaussian or Bernoulli distributions), which are often sub-optimal. The DL-based approach still retains the hardware benefits from the linear sensing process of CS. In contrast, most DL-based CS methods considered sensing matrix optimization during the training process. To further increase the compression ratio, various quantization approaches were adopted in DL-based CS methods. Sun et al. (2016) and Sun and Feng (2017) jointly optimized a binary sensing matrix and a reconstruction network simultaneously, outperforming traditional sensing matrices in terms of both recovery quality and computation time. Moreover, a non-uniform multi-bit quantizer for compressed measurements quantization was proposed, which outperformed the uniform quantizers for wireless neural recording, especially with low quantization bit-depth (Sun et al., 2016). In Sun and Feng (2017), the entries of the sensing matrix were quantized into one bit, thus reduced its storage requirement and boosted the sensing process. More recently, the training-free deep generative model has been successfully applied to AP compression and outperformed model-based and data-driven methods (Sun et al., 2021a).

The advantages of DL-based methods are the deep features extracted from the neural data, thereby avoiding manual designs of sparse dictionaries and yielding better reconstruction performance. However, there is still a lack of comprehensive understanding on the robustness and generalization capabilities of DL-based approaches. Also, the network may need to be retrained when recording and application scenarios are changed.

## 5. Remaining Challenges

Here we list the remaining challenges for CS-based wireless neural recording systems:

**Hardware Efficiency Toward Large-Scale Neural Recording**: Despite the initial success, the hardware cost of CS can still be costly for high-density wireless neural recording devices. Aggressive approaches on the construction of hardware-efficient sensing matrices and circuit-level efforts to further minimize the computational hardware and measurement storage elements are required.

**Sparse Representation for Raw Neural Signals**: So far, CS cannot be applied to raw neural signals. This mainly originates from the fact that it lacks the knowledge of sparse representation of raw neural signals (Pagin and Ortmanns, 2018), which causes reduced signal reconstruction accuracy and eventually low performance in spike detection and sorting accuracy. Current CS approaches require the knowledge of spike location and only perform well on spike segments. This also limits the analog CS front-end designs for raw neural signals to reduce the sampling rate. If such sparse representation exists, this could bring extra opportunities for the CS-based neural signal compression tasks.

**Overlapped Spikes and Misalignment**: Neural recording based on multielectrode technology measures the activities from thousands of neurons simultaneously; it is, therefore, highly likely to observe multiple spikes within the same spike window, which is also referred to as the overlapping spikes. Currently, overlapping spikes are excluded and there is limited success for the signal reconstruction of overlapping spikes using CS methods. If successful, state-of-the-art spikes sorting approaches (e.g., KiloSort, Pachitariu et al., 2016) can therefore be incorporated for accurate neural coding tasks. Moreover, accurate on-chip spike alignment is difficult and can be hardware demanding due to low signal-to-noise ratio, sampling jitter, and noise effects (Gibson et al., 2011). Therefore, CS algorithms should be robust in the presence of spike overlapping as well as misalignment.

**Real-Time Signal Reconstruction**: The increasing number of channels is an irreversible trend in neural recording applications, which also brings huge computational burdens on the real-time performance of CS reconstruction. Most multi-channel CS algorithms exploit the intra- and inter-channel correlations to improve the signal reconstruction speed (Sun et al., 2021b). Although the reconstruction efficiency is somewhat improved, the overall reconstruction accuracy decreases compared with the channel-by-channel reconstruction approach. Moreover, the channel correlation assumption may not always hold valid. Therefore, how to balance the reconstruction accuracy and speed remains a major challenge for CS-based large-scale neural recording applications.

## 6. Conclusion

This paper reviews the CS-based extracellular neural recording systems reported in the research literature in the past decade. CS has been widely considered to be a promising technique and actively studied for neurophysiology signal compression purposes. The key aspects of CS, i.e., the sensing matrix (both analog and digital CS encoder design), the sparse representation of neural signals (both APs and LFPs), and the corresponding signal reconstruction algorithms, are covered. In particular, the associated challenges are discussed in detail at different CS stages for different neural signal modalities. Despite its current progress, there remain several challenging topics needed to be resolved toward practical CS-based neural recording systems in the future.

## Author Contributions

Both authors contributed to manuscript planning, literature review and writing, and read and approved the submitted version.

## Conflict of Interest

The authors declare that the research was conducted in the absence of any commercial or financial relationships that could be construed as a potential conflict of interest.

## Publisher's Note

All claims expressed in this article are solely those of the authors and do not necessarily represent those of their affiliated organizations, or those of the publisher, the editors and the reviewers. Any product that may be evaluated in this article, or claim that may be made by its manufacturer, is not guaranteed or endorsed by the publisher.
